# Health-related confidentiality and consent among minors: Data on adult perspectives from Belgium and The Netherlands

**DOI:** 10.1016/j.dib.2022.108301

**Published:** 2022-05-22

**Authors:** David De Coninck, Koen Matthijs, Peter de Winter, Jaan Toelen

**Affiliations:** a Centre for Sociological Research, KU Leuven, Belgium; b Leuven Child and Youth Institute, KU Leuven, Belgium; c Department of Pediatrics, Spaarne Gasthuis, the Netherlands; d Department of Development and Regeneration, KU Leuven, Belgium; e Department of Pediatrics, University Hospital Leuven, Belgium

**Keywords:** Adolescents, Confidentiality, Consent, Medical decision-making, Minors, Parents

## Abstract

The data presented in this article provide one of the first large-scale insights on adult preferences for confidentiality and consent with regards to medical decision-making for minors. We collected data on these preferences through 12 hypothetical scenario's that were presented, for which each participant had to indicate if they would (not) follow the minor's preferences. Data regarding family communication, relationship quality, and sociodemographic characteristics were also collected. The data were collected through an online survey in September and October 2020, which yielded responses from 1000 Belgian and 1000 Dutch participants between 35 and 55 years of age. We selected this age range because it increased the chances that they had a child near the age of the fictional minor in the hypothetical cases. These data can be of interest for family researchers and/or health workers who want to explore adults’ perceptions regarding confidentiality and consent among minors.

## Specifications Table

**Table:** 

Subject	Sociology; Public Health and Health Policy
Specific subject area	Adults’ perceptions regarding health-related confidentiality and consent among minors
Type of data	Table
How the data were acquired	Online survey among adults aged 35 to 55 in Belgium and The Netherlands
Data format	Raw
Description of data collection	Being over the age of 34 and under the age of 56 in Flanders, the northern, Dutch-speaking region of Belgium, and The Netherlands, and residing in one of these regions at the time of the fieldwork (September through October of 2020).
Data source location	• Institution: KU Leuven • City/Town/Region: Leuven • Country: Belgium
Data accessibility	Repository name: Mendeley DataData identification number: http://dx.doi.org/10.17632/mxmt3cy5dx.1Direct URL to data: https://data.mendeley.com/datasets/mxmt3cy5dx/1

## Value of the Data

• The data presented can contribute to a better understanding of the ways in which adults (including a large number of parents) reflect on confidentiality and consent regarding health-related cases among minors in Belgium and The Netherlands.
• Researchers in family studies can benefit from these data because they highlight how these perceptions differ according to specific family configurations and what the role of stepparents in decision-making should be.
• Researchers in public health and practitioners can benefit from the case-based methodology that we applied to gauge confidentiality and consent preferences, as these are more grounded in reality than more abstract survey measures. Furthermore, the inclusion of cases regarding stepparents corresponds to a growing demand from practitioners to receive new insights on how to deal with alternative family configurations during consultations.
• The data presented are unique: very little large-scale survey data exists that map confidentiality and consent preferences regarding health-related decision-making among minors. Most of the current literature in this field is built on qualitative data. Our data allow for new insights in a highly relevant field that is rapidly evolving, and where researchers, practitioners and policy makers alike are looking for new insights.

## 1 Data Description

The quantitative data presented in this article were collected through an online survey. It was fielded to investigate adult preferences regarding health-related confidentiality and consent among minors. No funding was obtained for this study. With these data, we provide new insights into the preferences of adults regarding 12 different hypothetical cases. The need for new insights in this topic is great as survey data are rare, despite the growing diversity of family types in recent years [1,2]. Through this online survey, we collected quantitative data on preferences regarding health-related confidentiality and consent for minors, family communication, relationship quality, and sociodemographic characteristics for the adult population aged 35 to 55 in Belgium and The Netherlands. This age range was selected because the hypothetical cases mostly revolved around a (fictional) 15-year old adolescent and we wanted to maximize the chances that participants had a child near that age. We collected the data in cooperation with Bilendi, a Belgian polling agency, and selected the methodology for its cost-effectiveness in cross-country research. Respondents received an e-mail asking them to participate in a survey without specifying the subject matter, which was essential to avoid priming. Three weeks of fieldwork in September and October of 2020 resulted in a dataset of 2,000 respondents (1,000 per country). The cooperation rate was about 13%. While Table 1 shows the distribution of respondents by several socio-demographic characteristics, Table 2 presents mean scores of family communication and relationship quality indicators and the distribution of the answers on the 12 cases. In the associated data file, we included the full data set, codebook, and survey presented to participants [3].

**Table 1:** Descriptive overview of the sample (*N* = 2,000).

	Belgium	The Netherlands
In %		

Gender		
Male	48.7	35.6
Female	51.3	64.4
Age		
Between 35 and 44 years	42.4	36.5
Between 45 and 55 years	57.6	63.5
Marital status		
Unmarried, never married	13.6	12.6
Legally or de facto cohabiting	22.7	16.9
Married	52.3	55.1
Divorced	8.4	11.5
Separated	2.3	2.5
Widowed	0.7	1.4
Full-time job	73.0	40.8
Educational attainment		
Secondary education or lower	34.7	56.2
Tertiary education or higher	65.3	43.8
Migration background	4.6	14.0
Biological or adoptive child(ren)	65.6	72.6

Stepchild(ren)	14.7	16.3

In mean score	Belgium	The Netherlands

Financial deprivation	4.10 (1.11)	3.93 (1.13)

N	1000	1000

Note: Financial deprivation answer options ranged from 1 (very difficult to make ends meet) to 6 (very easy to make ends meet).

**Table 2:** Overview of answers on case-based scenario's and family relationship items (*N* = 2000).

	Belgium	The Netherlands
In %		

Case 1: Drunkenness		
Physician reports drunkenness to parents	86.0	80.2
Physician does not report drunkenness to parents	14.0	19.8
Case 2: STD		
Physician reports infection to parents	61.7	54.8
Physician does not report infection to parents	38.3	45.2
Case 3: Ultrasound		
Physician does not report reason for scan to parents	25.1	32.3
Physician reports reason for scan to parents	74.9	67.7
Case 4: Depression		
Physician does not report findings to parents	28.3	38.0
Physician reports findings to parents	71.7	62.0
Case 5: Placebo		
Physician does not prescribe placebo	63.0	63.5
Physician prescribes placebo	37.0	36.5
Case 6: Maxillofacial surgery		
Physician prevents surgery	30.6	33.5
Physician allows surgery to go on	69.4	66.5
Case 7: Migraine		
Physician does not prescribe medication	17.1	28.2
Physician prescribes medication	82.9	71.8
Case 8: ADHD		
Physician does not prescribe medication	35.0	38.1
Physician prescribes medication	65.0	61.9
Case 9: Test results		
Physician does not provide info to stepmother	16.1	30.6
Physician provides info to stepmother	83.9	69.4
Case 10: Vaccination		
Physician does not give the vaccine	32.1	45.6
Physician gives the vaccine	67.9	54.4
Case 11: Informed consent		
Physician does not allow stepfather to sign	29.7	46.7
Physician allows stepfather to sign	70.3	53.3
Case 12: Blood sample		
Physician does not take blood	18.1	35.2
Physician takes blood	81.9	64.8

In mean score	Belgium	The Netherlands

Family communication		
In my family we are satisfied about how we communicate with each other.	3.66 (0.89)	3.87 (0.86)
My family are good listeners.	3.80 (0.83)	3.90 (0.81)
In my family, we show our love to one another.	4.02 (0.85)	4.18 (0.78)
In my family we can ask each other what we want.	3.99 (0.83)	4.19 (0.78)
In my family we can talk about our problems with each other.	3.75 (0.89)	3.98 (0.82)
In my family we can discuss our ideas and convictions with each other.	4.03 (0.80)	4.17 (0.75)
In my family we honestly answer each other's questions.	3.97 (0.79)	4.10 (0.74)
In my family we try to understand each other's feelings.	4.00 (0.77)	4.11 (0.74)
In my family we rarely say negative things about each other when we are angry.	3.09 (1.02)	3.35 (1.01)
In my family we express our genuine feelings.	3.71 (0.88)	3.92 (0.83)
Relationship quality		
Current partner	6.08 (1.14)	6.21 (1.20)
Children from current relationship	6.46 (0.76)	6.49 (0.91)
Children from previous relationships	6.15 (1.28)	5.91 (1.66)
Children from partner from previous relationship	5.71 (1.54)	5.83 (1.59)

Note: * Family communication answer options ranged from 1 (do not agree at all) to 5 (fully agree). Family relationship quality answer options ranged from 1 (very bad) to 7 (very good).

## 2 Experimental Design, Materials and Methods

To identify respondents' preferences regarding confidentiality and consent, we presented twelve hypothetical cases: four on confidentiality, four on consent, and four on the role of stepparents in medical decisions. In the first eight cases, the fictional adolescent has received or requires medical treatment. This adolescent wishes that the parents are either not informed about the requested or required treatment, or that the treatment is continued despite parental opposition. For each case, respondents were asked to indicate their own preference. The age of the fictitious adolescent was set to 15 years because this is the age at which - in 18 of the 27 EU countries, including Belgium - adolescents are not considered legally competent, but can be found competent by a physician [4]. Below, we present an overview of the specific wording of each case, with answer options presented in Table 2. We also include further details on the scale on family communication.

## 3 Confidentiality

Case 1 (Drunkenness): Your son went to a party with friends and drank alcohol. Afterwards, he tripped and fell on the floor and unfortunately ended up with his hand in a glass shard. Because of this injury, he was taken to the emergency room where a doctor stitched the wound. Your son realizes that he will be in trouble when you (as a parent) will hear about the intoxication and asks the attending physician not to inform you about it.

Case 2 (STD): Your daughter has recently started a romantic relationship and has an annoying problem for which she goes to the general practitioner. They diagnose a sexually transmitted disease (STD), which is easy to treat without side effects. She asks the practitioner not to say anything to you (the parents) about this infection.

Case 3 (Ultrasound): You received a hospital bill in the mail this week for an abdominal ultrasound performed on your son. You ask about the reason for this, but your son will not say. You contact the general practitioner who made the request for this examination and ask for the reason for this examination.

Case 4 (Depression): In recent weeks you have noticed that there is a problem with your daughter: she sleeps poorly, always retreats to her room, doesn't talk to her friends anymore, is often in a gloomy mood and eats badly. You know that your daughter went to the general practitioner for this a few days ago, but you do not know what was discussed there.

### 3.1 Consent

Case 5 (Placebo): Your son suffered from problems falling asleep a few months ago. The general practitioner then prescribed melatonin, a drug to facilitate falling asleep, with good effects. You are convinced that this is more likely because your son believes too strongly in the medication. You make an appointment with the practitioner asking him to prescribe a placebo. You want to swap these pills with the melatonin without telling your son. In this way, you want to help him stop taking that medication.

Case 6 (Maxillofacial surgery): Your daughter has an appointment with an oral and maxillofacial specialist in connection with her teeth. She has a so-called underbite. The lower jaw is set back too much, and this leads to bullying by peers. The doctor says that the only way to get the teeth completely straight is surgery, which involves sawing through the lower jaw. Your daughter values her looks and wants this surgery. She hopes that this will stop the bullying. However, you think your daughter looks good and think surgery is dangerous and unnecessary.

Case 7 (Migraine): Your son has been suffering from migraines for about a year and is very much affected by them three times a month. He would like to do something about this, as these severe headaches interfere with school, sports and hobbies. Your general practitioner suggests a drug treatment to decrease the headache, but this medication sometimes has side effects. Your son thinks this is a good solution, and would like to try it. However, you feel that a 15-year-old adolescent should not be taking long-term treatment with medication, and you do not agree with this treatment method. Unfortunately, there are no other effective treatments available.

Case 8 (ADHD): You and your daughter consult the general practitioner because of problems at school. During the most recent meeting with teachers, you were told that there were some comments about disruptive behavior in class. In addition, the assessments/grades on the most recent report card are worse than last year. Your daughter has an attention disorder (ADHD) and has been taking medication for this for the past few years to improve her focus, with a good effect on behavior and grades. However, she tells the doctor that she no longer wishes to take this medication because of the 'inhibited feeling' associated with it. However, you realize the importance of good school results for the future of your daughter.

### 3.2 Role of Stepparents

Case 9 (Test results): A father consults the general practitioner with his own 6-year-old daughter because of fever and abdominal pain. The doctor suspects a bladder infection and sends a urine sample from the girl to the laboratory for analysis. In the afternoon, the stepmother visits with the girl (her stepdaughter) to discuss the results because the father has to work.

Case 10 (Vaccination): A stepmother consults the general practitioner with her stepson of 6 years old because of constipation. Two weeks ago, the father had already visited the general practitioner for this problem and for a scheduled vaccination. At that time, it was decided to start a treatment (fiber in powder form), the consultation today was among other things to see if this has improved. Because the boy was also sick two weeks ago, the planned vaccination was postponed until today. The doctor says that he wants to give the vaccine today, but the stepmother - who is worried about vaccines and possible side effects - says that the doctor cannot give the vaccine.

Case 11 (Informed consent): A stepfather accompanies his 6-year-old stepson who comes to the hospital to have a gastroscopy. This examination was discussed at a previous consultation, where both parents were present, and scheduled after general agreement. Because this examination will be done under sedation (a type of drug-induced "intoxication"), a parental consent document must be signed today (informed consent form or "informed consent").

Case 12 (Blood sample): A stepfather comes to the emergency department with a 6-month-old baby because of a high fever, the (biological) mom herself is sick in bed at home. Because the physician wants to rule out a serious infection, a blood sample is taken. But because the child is feverish, the first injection attempt fails. When the doctor wants to perform a second injection attempt, the stepfather refuses and wants to take the child back home.

### 3.3 Family Communication

With regards to family communication, we included the Family Communication Scale that was developed by Olson and Barnes [5]. This measure consists of 10 items that assess perceptions on the way in which family members communicate with each other on a daily basis. Answer options range from 1 (do not agree at all) to 5 (fully agree). Each item was coded in the same direction, simplifying the creation of potential composite measures on family communication through mean calculation based on this scale.

## Ethics Statements

Informed consent was obtained from all participants prior to participation in the study. Ethical approval was obtained from the KU Leuven Social and Societal Ethics Committee (G-2020-1670-R4).

## CRediT authorship contribution statement

**David De Coninck:** Conceptualization, Methodology, Software, Writing – original draft. **Koen Matthijs:** Conceptualization, Methodology, Writing – review & editing. **Peter de Winter:** Conceptualization, Methodology, Writing – review & editing, Supervision. **Jaan Toelen:** Conceptualization, Methodology, Writing – review & editing, Supervision.

## Declaration of Competing Interest

The authors declare that they have no known competing financial interests or personal relationships that could have appeared to influence the work reported in this paper.

## Data Availability

Health-related confidentiality and consent among minors: Data on adult perspectives from Belgium and The Netherlands (Original data) (Mendeley Data).
